# Sustainability effect of geogrid reinforced tire-shred sand mixtures on the load pressure-settlement response of shallow footing

**DOI:** 10.1016/j.heliyon.2022.e11743

**Published:** 2022-11-17

**Authors:** Hussein Ahmad

**Affiliations:** Faculty of Civil Engineering, Department of Geotechnical Engineering, Tishreen University (TU), Latakia, Syria

**Keywords:** Geogrid wraparound, Tire-shred, Fine sand, Bearing capacity, Settlement

## Abstract

This study aims to observe the possibility of using tire shreds as sustainable waste materials by including a geogrid wrap-around sheet to improve the response of load-settlement of the sandy bed. Therefore, a series of physical modeling tests were conducted to investigate the behavior of tyre-shred-geogrid reinforced sand under static plate loading. Experimental tests on tire-shred sand reinforced with geogrid sheets were carried out in the first step. Next, laboratory tests were conducted on tire-shredded sand with geogrid wraparound sheets, and the results were compared with the initial experiment. In terms of the parameters studied, the total depth of the tire-shred with planar and wraparound layers, the length and overlaps of the wraparound layers, and the space between the wraparound geogrid sheets were analyzed. According to the results, the inclusion of tire-shields in reinforced soil foundations with wraparound geogrid layers significantly improved the load-bearing capacity. Additionally, both planar and folded geogrid sheets had bearing capacity ratios greater than 3 and 4.5. The carrying capacity of soil-tire shred reinforced with inclusion of geogrid sheet is superior to that of geogrid-reinforced soil without tire shred.

## 1 Introduction

Shallow footings are typically developed on soil foundations with insufficient bearing capacity, due to either poor soil carrying capacity or increased footing settlements. One solution given to this problem is to improve the soil's strength qualities through the use of a reinforcing method, which helps to increase the soil’s carrying capacity and reduce pressures dissipated from the base of the shallow footing into the soil mass. Therefore, infrastructural development is difficult because of a lack of viable land. As land with desirable geotechnical properties is typically expensive and scarce, engineers use various methods of stabilizing, grouting, and compacting existing land for development. While these techniques are sometimes effective, they can be time-consuming, cumbersome, and expensive. As an alternative to building expensive deep footings, geosynthetics can be used.

In numerous studies, investigators have used experimental studies and numerical methods to investigate the effects of different factors and parameters on strengthened soil performance [1, 2, 3, 4, 5, 6, 7, 8, 9, 10, 11, 12, 13, 14, 15, 16, 17, 18]. For the first time, a series of experimental studies were carried out by Binquet and Lee [1] to improve the carrying capacity of strip footings using metallic strips. After that, several studies concluded that reinforcement within a specific embedment depth can increase the load-settlement response and peak load carrying capacity of the soil but had little effect on the overall strength [2, 3, 4, 5, 6, 7, 8, 9, 10, 11, 12]. Depending on the findings of the previous literature research [13, 14, 15, 16, 17, 18], the settlement features and ultimate carrying capacity of the soil foundation can be improved by inserting geosynthetic reinforcements into the soil up to a certain embedded depth, beyond which no significant improvement gives. There have been numerous developments over geosynthetic sheets for improving the soil bearing capacity, inclusions in geocell mats [19, 20], the behavior of geocells with/without geogrid-reinforced granular soil overlaid a stiff clay bed Biswas et al. [21], and the impact of wraparound geosynthetic sheets on the load-settlement behavior of shallow footings [22, 23, 24, 25].

Furthermore, some techniques are becoming increasingly popular, including random tire-shred sands (RTSSs). In this method, the soil is combined with discrete tire shreds and compacted in the field. The urban services organization in Iran has already dealt with the effects of waste tires on the environment. According to Fathi and Farahani [59], the use of tires for transportation will reach 800.000 Tons annually in 2026. According to Soheili [60], tires are currently used for 350.000 tons of fuel a year, which could be employed as an alternative fuel in the cement industry. For this study, the environmental benefits and effects of waste tires in cement plants were analyzed. According to available statistics, Iran uses 200,000 tons of tires each year, or 10 million tires. Approximately 20% of waste tires are recycled in Iran, while the other 80% are landfilled. It is challenging to dispose of waste tires since they have a large volume compared to their weight. The benefits of using waste tires for geotechnical applications include their low unit weight, low level of earth pressure, energy efficiency, vibration dampening, and low cost. Previous studies have been indicated that waste tires can increase the carrying capacity of granular soil when used under shallow footings [51, 63, 69].

Using laboratory plate load tests, Yoon et al. [61, 62] indicated that the benefit use of waste tyre sidewalls as a reinforcement inclusion within sandy bed. Based on the results, using one planar geosynthetic sheet in the medium-dense sandy soil is recommended to decrease settlements by half and to improve carrying capacity by two. It is reported that the optimize quantity of inclusion of tyre shred reinforcement for footings is approximately 40% by volume, after which the carrying capacity decreases [54]. A bearing capacity ratio of up to 3.9 was observed in the model footing tests. Moghaddas Tafreshi and Norouzi [55] suggested using 5% (volume) tire chips and a depth of 0.25B (in which B denotes the footing width). An improvement in carrying capacity of 2.68 times was found at a settlement ratio (s/B%) of 5%. According to Naval et al. [56], shallow footing used to rest on reinforced sand with waste tyre fibers have shown that even sands with a high relative density can be improved in terms of carrying capacity. The BCRs up to two were observed with fiber content of 0.75% and at embedded depth 1.5B. The behavior of granulated rubber reinforced sand was studied [52]. The tire content can be as much as 10% by weight, the reinforcement depth can be up to 1B, and the tire width can be up to 5B depending on the tire. An increase of 1.55 BCRs were found with some tires. Waste tire chips have been used as a substitute for sand [63], and their optimum content was observed to depend on applied strains. The recommended tire chip contents for low and high strains are 20% and 40%, respectively. At low and high strains, BCRs reached 2.82 and 7.8, respectively.

Although several studies have examined the impact of different geogrid parameters on the performance of strip footing resting on reinforced fine dense sandy soil, there are limited studies that describe the failure behavior of tire-shred geogrid soil systems, especially the failure of this system with folded geogrids. To date, the relationship between strip footing failure mode and the response of wraparound geogrid sheets has not been thoroughly examined and analyzed. In this respect, it is important to look at the failure modes of strip footing and the effect of wraparound geogrid sheets. Based on a review of the literature [51, 52, 53, 54, 55, 56, 57, 58, 59, 60, 61, 62, 63, 64, 65, 66, 67, 68, 69], these studies have investigated the utilize of waste tyre shreds (size 10 mm) as an inclusion of reinforcement elements underneath shallow strip footing. Early studies tended to focus on shreds or granulated forms of tires. A smaller number of studies have examined tire chips. There is also little evidence on the load pressure-settlement behavior of tire chips, as earlier studies examined the load-settlement behavior of uniform-sized tire chips. In contrast to previous TSS studies, in the present study, we have investigated both improved bearing capacity at low settlements (s/B (%) = 2%) and high settlements (s/B (%) = 10%). Furthermore, the combined application of folded reinforcement with/without tire-shielding was compared.

## 2 Methodology and material used

To illustrate the performance of the suggested approach, laboratory tests are required. The features of the current experimental investigations are presented in the following section.

### 2.1 Soil material used

**Figure 1:**
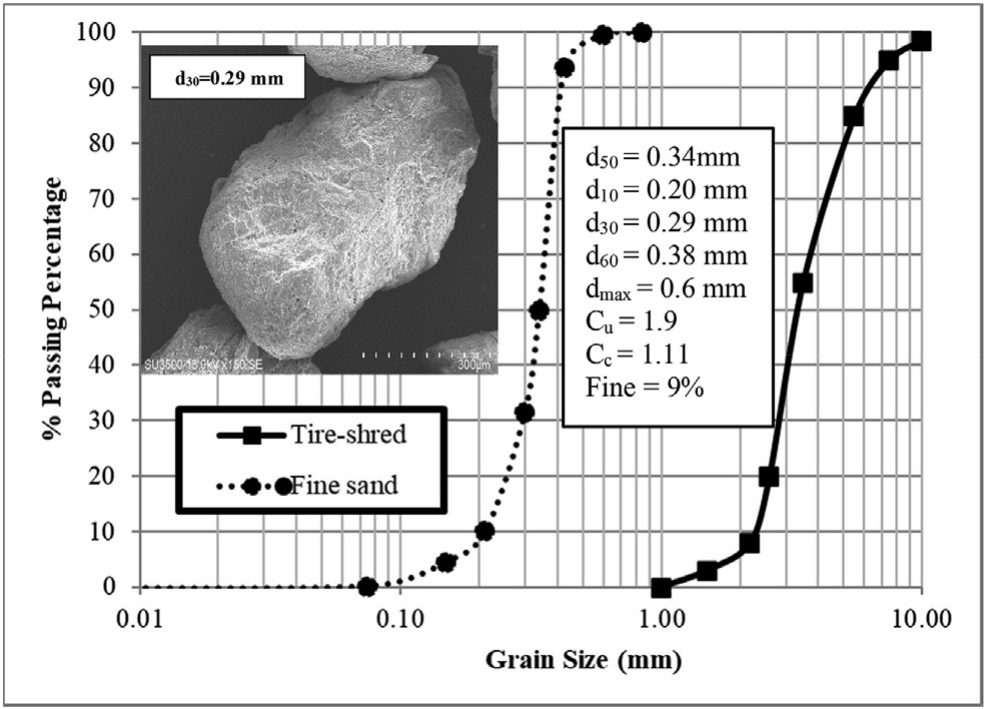
Particle size distribution analysis.

**Figure 2:**
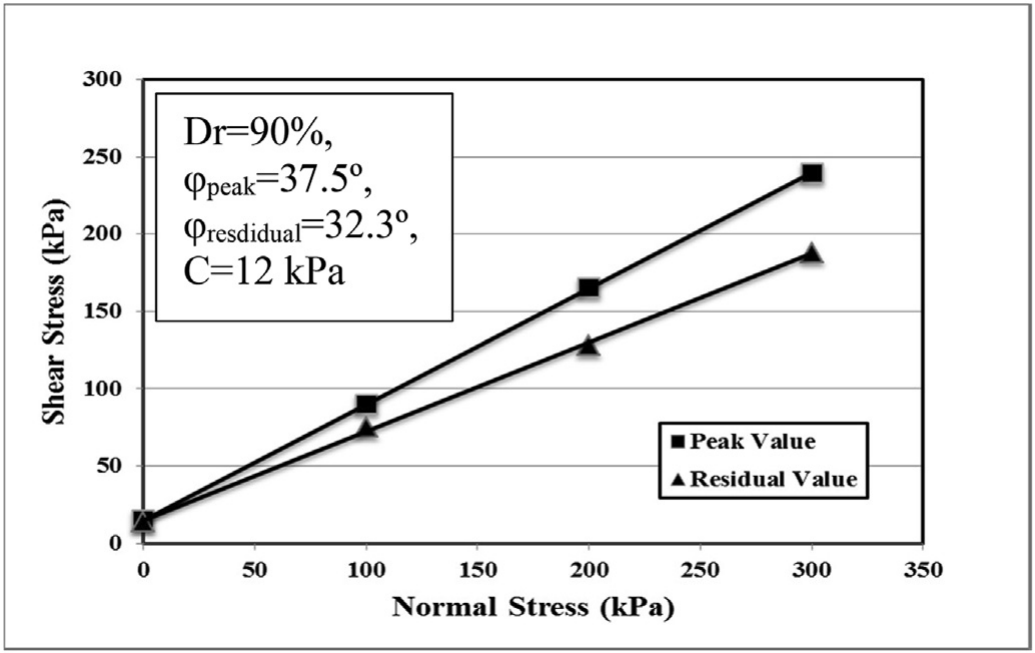
Mohr–Coulomb envelope curve on Firozkoh fine sand.

The fine-grained sandy soil Firozkoh (code no. 151) was utilized for the experimental tests [25, 26]. The sandy soil was sampled in the mountainous region of Tehran in northeastern Iran. The standard ASTM D422 [27] of soil classification was utilized to demonstrate the particle size analysis using sieves of various mesh openings. Based on the results that cited in Table 1 and Figure 1, by using the standard of the Unified Soil Classification System (USCS), the soil used is described as poorly graded sandy soil (SP). The determination of the dry unit weight of the soil was guided by ASTM D2049 [28], and the minimum dry unit weight (γ_dmin_) of the sandy soil used is 14.09 kN/m^3^. By using the vibrating test table, the maximum dry unit weight (γ_dmax_) in the same mold size was determined, and γ_dmax_ of 16.61 kN/m^3^ was obtained considering the specific gravity of Gs = 2.68. The direct shear test is conducted by using a square mold of 100 × 100 mm based on ASTM D5321 [29] at 90% relative density under dry conditions. The magnitudes of the basic parameters of fine sand soil (C, φ_risdual_ and φ_peak_) are shown in Table 1 and Figure 2. The oedometric modulus was obtained from a one-dimensional test under dry conditions.

**Table 1:** The soil properties used in the physical model.

Parameter	Magnitudes
Cohesive, C (kPa)	12
Specific gravity, G_s_	2.68
Residual friction angle, φ_residual_ (°)	32.3
Peak friction angle, φ_peak_ (°)	37.5
Maximum dry unit weight, γ_dmax_ (kN/m^3^)	16.61
Minimum dry unit weight, γ_dmin_ (kN/m^3^)	14.09
Dry unit weight used, γ_d_ (kN/m^3^)	16.46
Relative density, Dr (%)	90
Mean particle size, d_50_ (mm)	0.34
Effective particle size, d_10_ (mm)	0.20
Maximum particle size, d_max_ (mm)	0.60
Minimum particle size, d_min_ (mm)	0.01
Coefficient of curvature, c_c_	1.11
Coefficient of uniformity, c_u_	1.90
Oedometric modulus, E_oed_ (kPa)	30000
Fine pecentage	9

### 2.2 Geogrid reinforcement

**Figure 3:**
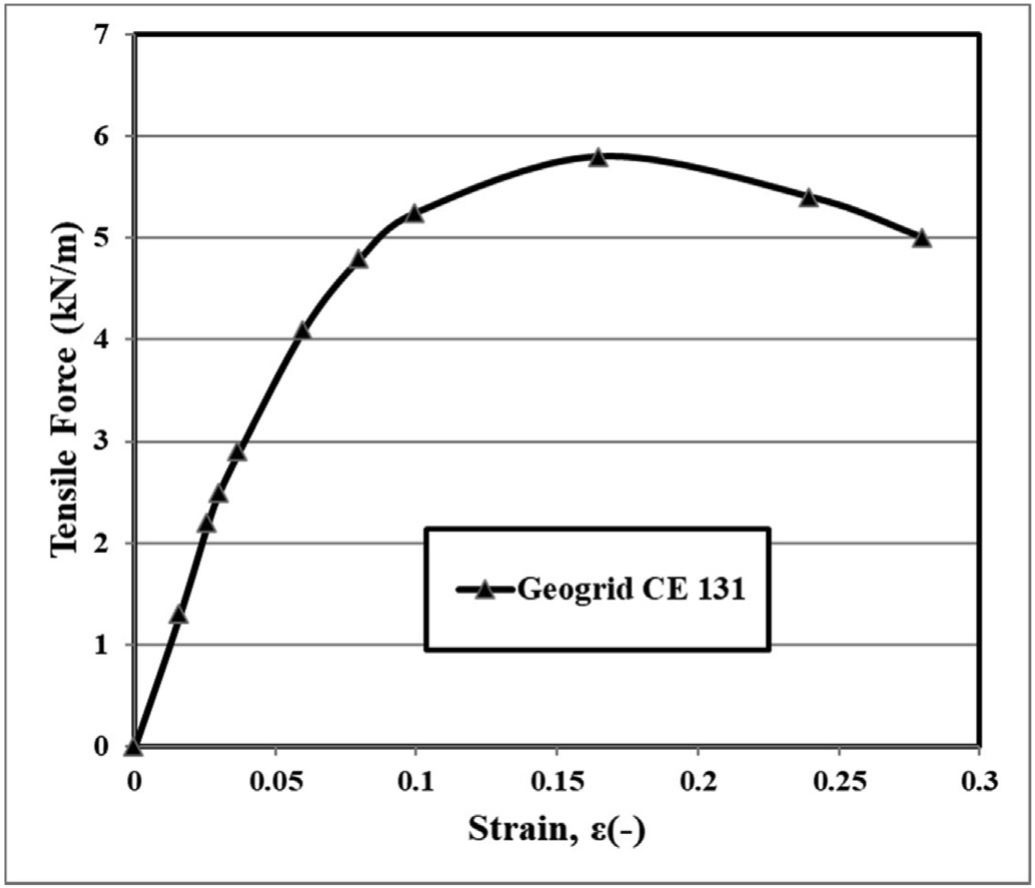
Tensile force-strain diagram of geogrid CE131.

One type of geogrid called CE131 was used as polymer HDPE in this study, which was manufactured by the Iranian company Ahmad et al. [26]. The unit weight, thickness, and mesh aperture size denote 0.70 kg/m^2^, 5.2 mm, and 27 mm, respectively, of this type of geogrid. The maximum tensile force and extension at maximum load were found to be approximately 5.8 kN/m and 16.5%, respectively. The geogrid used has the similar tensile strength. Figure 3 demonstrates the strain-tensile force diagram of this geogrid. The physical features of the geogrid used are determined due to an isolation test according to ASTM D 4595 [30]. In this research, the ultimate tensile strength of the geogrid is relatively low because the laboratory tests have been conducted by using the small-scale physical modeling. When a small footing width is used, it is suitable to utilize geogrid reinforcement with a low tensile strength and small aperture sizes to allow the interaction between the soil and geogrid used to achieve good performance.

### 2.3 Tyre-shred used

According to the previous literature [64, 65], the inclusion of tyre shred can significantly influence on the carrying capacity of the sandy soil bed. Geogrid-reinforced soil was analyzed with sand that contained tire shreds. Figure 1 illustrates the analysis of particle size distribution curve of the tyre shred. The specific gravity of the tyre shred grains is approximately 1.16, and their modulus of elasticity is 3.27 MPa. The smallest particle size (D_10_), the mean grain size (D_50_), and the largest particle size D_100_ are 2.2, 4.6, and 11.4 mm, respectively (ASTM D6270-17). After ensuring that no steel or cord was present in the particles, they were prepared from recycled truck tires.

In the laboratory, a series of six direct shear experiments (DSE) were conducted to determine the geogrid-tire shred interaction as indicated by Tanchaisawat et al. [70]. In the lower part of the shear specimen test, the tire shred–sand mixture was poured and compacted to achieve to the required thickness. As known in the small-scale of the direct shear test, the failure shear plane denoted this horizontal surface. Then, on this plane, one geogrid layer of aperture size 100 × 100 mm was used. In the next stage, the upper shear specimen test with its roller bearings was put directly above the geogrid sheet. The test procedure was conducted at three vertical normal stresses of 100 kPa, 200 kPa, and 300 kPa. As a result of the direct shear test with specimen test of 100 mm × 100 mm, the interior friction angle of the tyre shred-sandy soil mixture only was determined to be 31°, whereas that of the mixture interaction of tire shred-sandy soil with the geogrid layer was 30°.

### 2.4 Model foundation

The reduced scale modeling of the shallow foundation was carried out utilizing a single strip foundation made of rigid steel plates. The thickness of the strip foundation takes 2 cm; therefore, it considers as a rigid solid plate that is not subject to the bending impact. The width of this strip footing is 10 cm. The directional length of the strip foundations (39 cm) is similar as that of the width of experimental sandbox. By gluing a coarse layer of sand, the surface of the footing base is roughened with epoxy adhesive to get an uniform roughness in the whole experiments.

### 2.5 The test procedure and preparation

**Figure 4:**
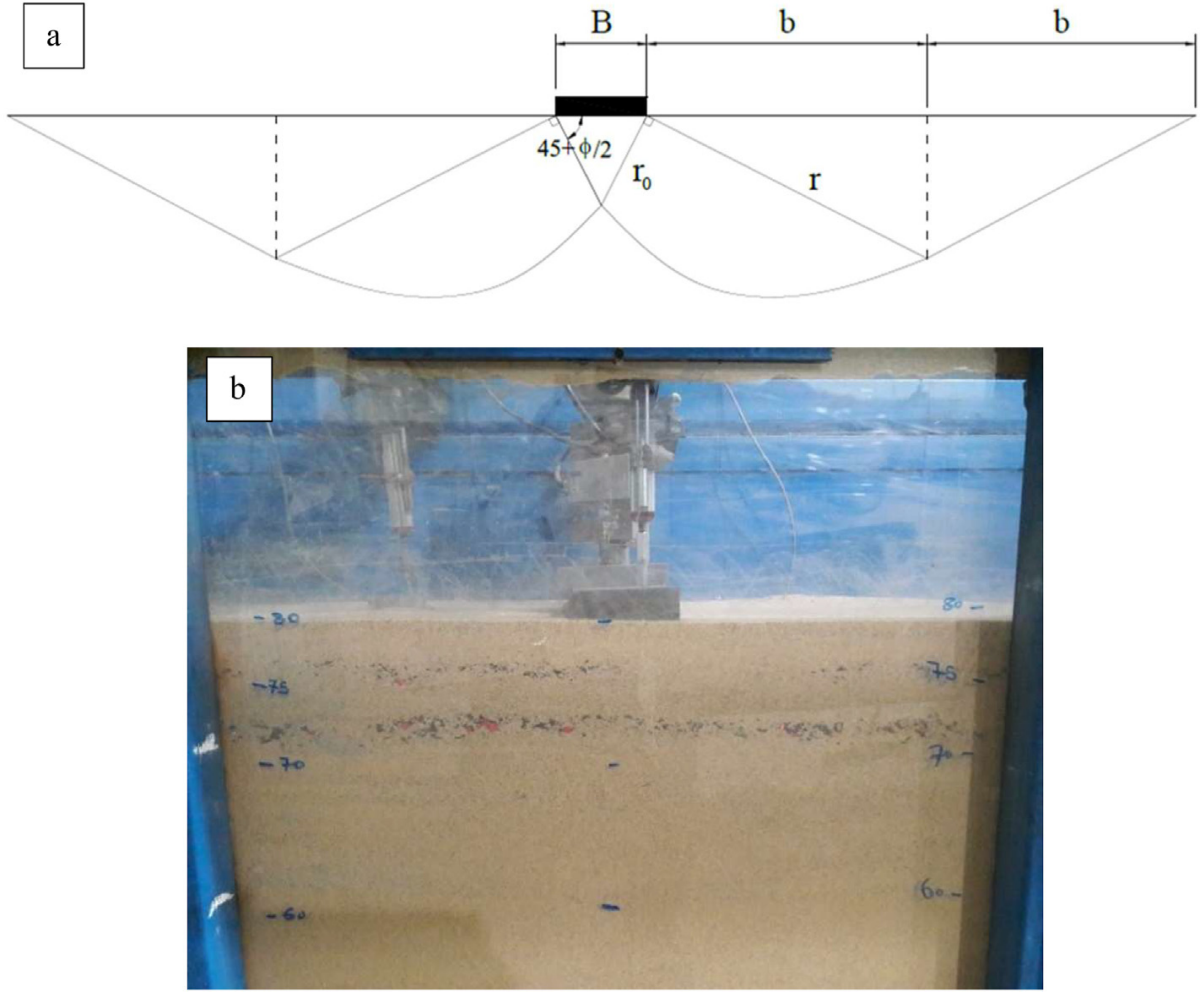
(a) Boundary effect on the mode failure and (b) image of a laboratory model test.

A large, cubic box manufacture of steel by these following dimensions of 80 cm height, 40 cm width and 160 cm length were utilized for the physical model tests. Due to the conditions associated with the plane strain issue, the horizontal plane of the sandbox was selected. The ratio L_sandbox_/B of 16 between the model length and foundation width is deemed sufficient such that its lateral dimensions are not interfered with by failure surfaces. Therefore, the dimensions length of the sandbox is too large, and the boundary effect is negligible. For the sandbox floor, 800 mm is the height of the bottom. There were no differences between the widths of the box and the foundation length. The study of the boundary effect on the mode failure is investigated as follows as demonstrated in Figure 4a. The shape of failure mode of the soil bed depends not only upon the side length of the boundary of sandbox but also upon the thickness of soil underneath a strip footing used.

The rotational shear failure become more prominent as the sand thickness increased. The embodiment depth of the triangular region beneath the base of footing denotes as u = B/2 × tan (45+φ/2) = 0.5B × tan(φ) for the strip footing plate resting on the sand employed in this research. If φ = 45 + φ/2 = 45 + 0.5 × 37.5° = 63.75° and B = 10 cm, then u = 1.014B = 10.14 cm. If three layers of geosynthetics have been used, then the total depth underneath the strip footing is d = 3u = 3.014B. As a result, the total height of the sandbox is H = d + 3.8B = 6.74B = 67.4 cm. Therefore, the effect of the soil height become negligible when the bottom base of the sandbox was very far away from the base of strip footing used, as indicated by Chang et al. [71]. On the other hand, in fine sandy soils, the rupture mechanism is calculated in Eq. (1) with a friction angle of 37.5° according to the effect of a rigid boundary, as follows:

(1) $$r_{0}=\frac{B}{2\times \cos\left(45+\frac{\emptyset }{2}\right)}=1.13B$$

The log radius spiral r is determined by Eq. (2):

(2) $$r=r_{o}e^{0.5\pi \;\tan\emptyset }=3.77B$$

The spacing from the edge of the footing used, b, at which zone II is done, is a function of the interior frictional angle of the soil and the log spiral r, as presented in Eq. (3):

(3) $$b=r\;\cos\left(45-\frac{\emptyset }{2}\right)=3.38B$$

The total width of the sandbox is B_tot_ = 2 × (0.5B+2b) = 14.5B = 145 cm.

Based on the assumption that the strip foundation model width is 10 cm in the studies analyzed in this theme, the rupture bandwidth is approximately 145 cm. As a final step, these dimensions 1600 × 100 × 500 mm of test tank were employed to evaluate the soil carrying capacity underneath the strip footing with using reinforcements.

Additionally, the base had its length diminished by 5 mm from each side, which prevented it from contacting the sidewall of the test tank. As a loading system, an air compressor equipped with a pneumatic cylinder was employed to provide an air pressure of nearly 10 bars. This ensures a uniform periodic loading of soil samples by applying pressure to the sample. Each soil test was performed with a 5-ton force jack to measure the edge tolerance of the soil. To measure the settlements in the foundation, three linear variable differential transformers (LVDTs) are employed. The load cell is employed to calculate the applied incremental force. ASTM D 1196 [31] was followed for the test procedure, where applied load measurements were taken continuously for 3 min until a settlement rate of 0.03 mm/min was reached.

To construct the specimen with uniformly placed soil throughout the sandbox, a technique known as rain sand was used [32]. Via several tests in an aluminum mug, the preferred unit weights were calculated by calibrating the sand particle fall velocity and fall height. The weight of each soil piece was determined using a soil tamper. The approximate unit weight of dense sand used at relative density of Dr = 90% are 16.46 kN/m^3^. The relative density of fine sand inside the geogrid wrap-around sheet is controlled employing the small cans of known volume put at different locations in the sandbox to compute the unit weight and relative density achieved through the tests. The relative density of the cans was within 1.5% of their confidence level. Loose to medium textured soils have been the subject of previous experiments. The lack of information data and laboratory findings on dense sandy soil makes it necessary to conduct a high compaction ratio study to demonstrate how the soil behaves. This technique is easy to use in a few limited practical conditions. First, the soil bed was prepared, and the total embedment depth was determined. Then, the sandy soil was compacted at a relative density of 90%, after which folded geogrid layers were placed in the real situation. The surface at this depth is leveled by compacting it and again placing another layer followed by repeating the previous steps. A schematic of the studied system is illustrated in Figure 4b.

**Figure 5:**
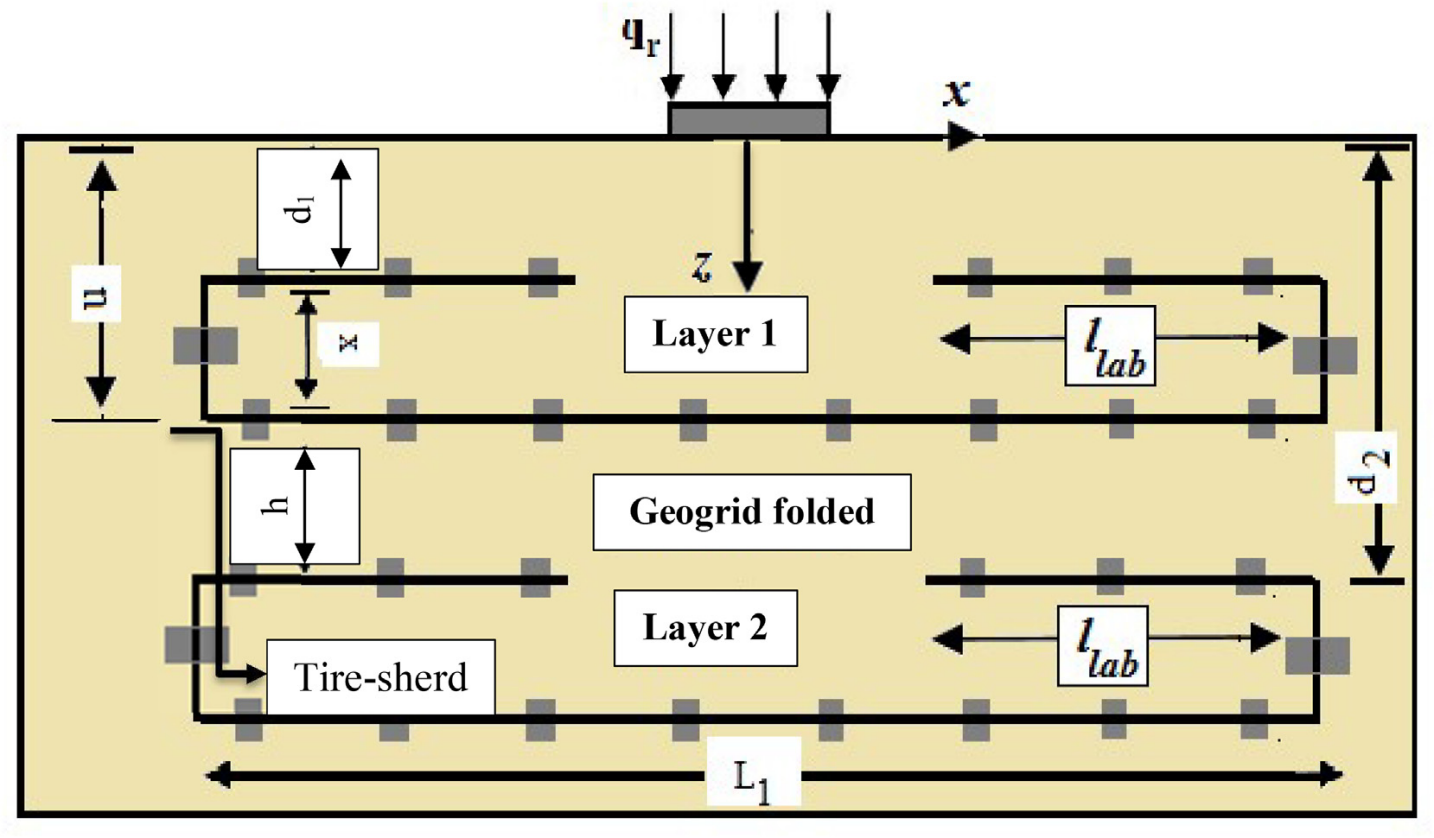
Layout of reinforcing folded arrangement.

Figure 5 summarizes the explanations of the model tests that are incorporated into the results for folded geogrids. Table 2 schematically presents all the experimental tests conducted in this work for a folded geogrid. In Table 2, l/L refers the use of the wraparound geogrid with lap length equal to zero. l/L = - refers to the wraparound geogrid that is not used, which means the soil is in an unreinforced state. l/L = planar refers to only a geogrid planar sheet.

**Table 2:** Experimental setup.

Tests No.	Form of reinforcement	N	Placement of geogrid	h/B	*l*/L
1	Unreinforced sand∗	0	-	-	-
2	Tire-sherd-sand∗	0	-	-	-
3	Semifolded∗	1	d = 0.2B, D = 0.3B	-	0.25
4	Semifolded	1	d = 0.2B, D = 0.3B	-	0.5
5	Semifolded	1	d = 0.2B, D = 0.3B	-	0.75
6	Full-folded∗	1	d = 0.2B, D = 0.3B	-	1.0
7	Semifolded	1	d = 0.1B, D = 0.3B, x_1_ = 0.2B	-	0.5
8	Semifolded	1	d = 0.2B, D = 0.4B, x_1_ = 0.2B	-	0.5
9	Semifolded	1	d = 0.3B, D = 0.5B, x_1_ = 0.2B	-	0.5
10	Semifolded	1	d = 0.4B, D = 0.6B, x_1_ = 0.2B	-	0.5
11	Semifolded	1	d = 0.5B, D = 0.7B, x_1_ = 0.2B	-	0.5
12	Semifolded	2	d = 0.2B, D = 0.8B, x_1_ = 0.2B	0.0	0.5
13	Semifolded	2	d = 0.2B, D = 1.2B, x_1_ = 0.2B	0.2	0.5
14	Semifolded	2	d = d_opt_, D = D_opt_, x_1_ = 0.2B	0.4	0.5
15	Semifolded	2	d = d_opt_, D = D_opt_, x_1_ = 0.2B	0.6	0.5
16	Semifolded	3	d = 0.2B, D = 1.2B, x_1_ = 0.2B	0.2	0.5
17	Semifolded-tire-sherd	1	d/B = 0.3, D/B = 0.6, x_1_ = 0.3B	-	0.25
18	Semifolded-tire-sherd	1	d = 0.3B, D = 0.6B, x_1_ = 0.3B	-	0.5
19	Semifolded-tire-sherd	1	d = 0.3B, D = 0.6B, x_1_ = 0.3B	-	0.75
20	Full-folded-tire-sherd	1	d = 0.3B, D = 0.6B, x_1_ = 0.3B	-	1.0
21	Semifolded-tire-sherd	1	d = 0.2B, D = 0.4B, x_1_ = 0.2B	-	0.75
22	Semifolded-tire-sherd	1	d = 0.3B, D = 0.6B, x_1_ = 0.3B	-	0.75
23	Semifolded-tire-sherd	1	d = 0.4B, D = 0.8B, x_1_ = 0.4B	-	0.75
24	Semifolded-tire-sherd	1	d = 0.5B, D = 1.0B, x_1_ = 0.5B	-	0.75
25	Semifolded-tire-sherd	2	d = 0.2B, D = 0.8B, x_1_ = 0.2B	0.0	0.5
26	Semifolded-tire-sherd	2	d = 0.2B, D = 0.8B, x_1_ = 0.2B	0.2	0.5
27	Semifolded-tire-sherd	2	d = 0.2B, D = 1.0B, x_1_ = 0.2B	0.4	0.5
28	Semifolded-tire-sherd	2	d = 0.2B, D = 1.2B, x_1_ = 0.2B	0.6	0.5
29	Semifolded-tire-sherd	3	d = 0.2B, D = 1.2B, x_1_ = 0.2B	0.2	0.5

N = number of geogrid sheets, x_1_ = thickness of wraparound geogrid sheet, d = embedment depth of lap component, D = the total embedment depth of geogrid sheets, h = vertical spacing between wraparound geogrid sheets, *l* = lap and overlap lengths, L = planar or wraparound geogrid sheet length, u = the first geogrid embedment depth, B = width of footing, and signs (∗) refer to repeated any test twice.

## 3 Results and discussion

### 3.1 Load-settlement response

A series of plate load tests were conducted on strip footings over geogrid-reinforced dense fine sand beds at the relative density of 90%. According to the report, the geogrid wraparound was primarily tested to assess its effects on the load-settlement behavior of strip footings over sand beds of fine grain. The parameters investigated were the carrying capacity for each experiment test and the strip footing settlement (s) normalized to footing width (B). Based on the bearing capacity-settlement diagram, an explanation of the effect of load testing is given. On the physical model shown in Figure 4, several 29 experimental tests were conducted to investigate the behavior of the strip footings on the soil under plane strain conditions. 4. Whenever a failure is detected, there is the continual introduction of vertical load up to the final settlement magnitude. The induced settlement of the foundation is given in millimeters, and the resulting curves are plotted by the applied pressure and settlement ratio (s/B%). Measurement of the effect of reinforced soil on strip footings was performed using a dimensionless factor (BCR). The parameter BCR depicts the ratio of the final carrying capacity of the soil with geogrid reinforcement to the final carrying capacity of soil without reinforcement (Eq. (4)), according to Badakhshan and Noorzad [17] and Xu et al. [33].

(4) $$BCR=\frac{q_{u\;\left(reinforced\right)}}{q_{u\;\left(unreinforced\right)}}$$

In this study, a new parameter can be defined as the increased bearing capacity ratio (BCR_I_), is introduced. Eq. (5) identifies the ratio of the peak bearing capacity in a geogrid-tire-shred reinforced soil to that in a non-tire-sherd addition (i.e., planar or wraparound) reinforced soil at a given settlement.

(5) $${BCR}_{I}=\frac{q_{u\;\left(reinforced-tire\right)}}{q_{u\;\left(planar-wraparound\right)}}$$

where q_u_ denotes the final carrying capacity.

In this work, the thickness of the tire-sherd mixed with fine sand is considered to be 2B and constant at all experimental tests. Mittal and Gill [63] recommended using two times the footing width of the tire-sherd layer thickness mixed in sandy soil at the same relative density of the soil bed.

### 3.2 Effect of single geogrid folded sheet with tire-shred

Soil reinforcement beneath shallow foundations has rarely been reinforced using geogrid wraparound techniques. Some advantages can be gained from using quality-controlled geogrids as a reinforced soil bed.

#### 3.2.1 Effect of upper lap length

**Figure 6:**
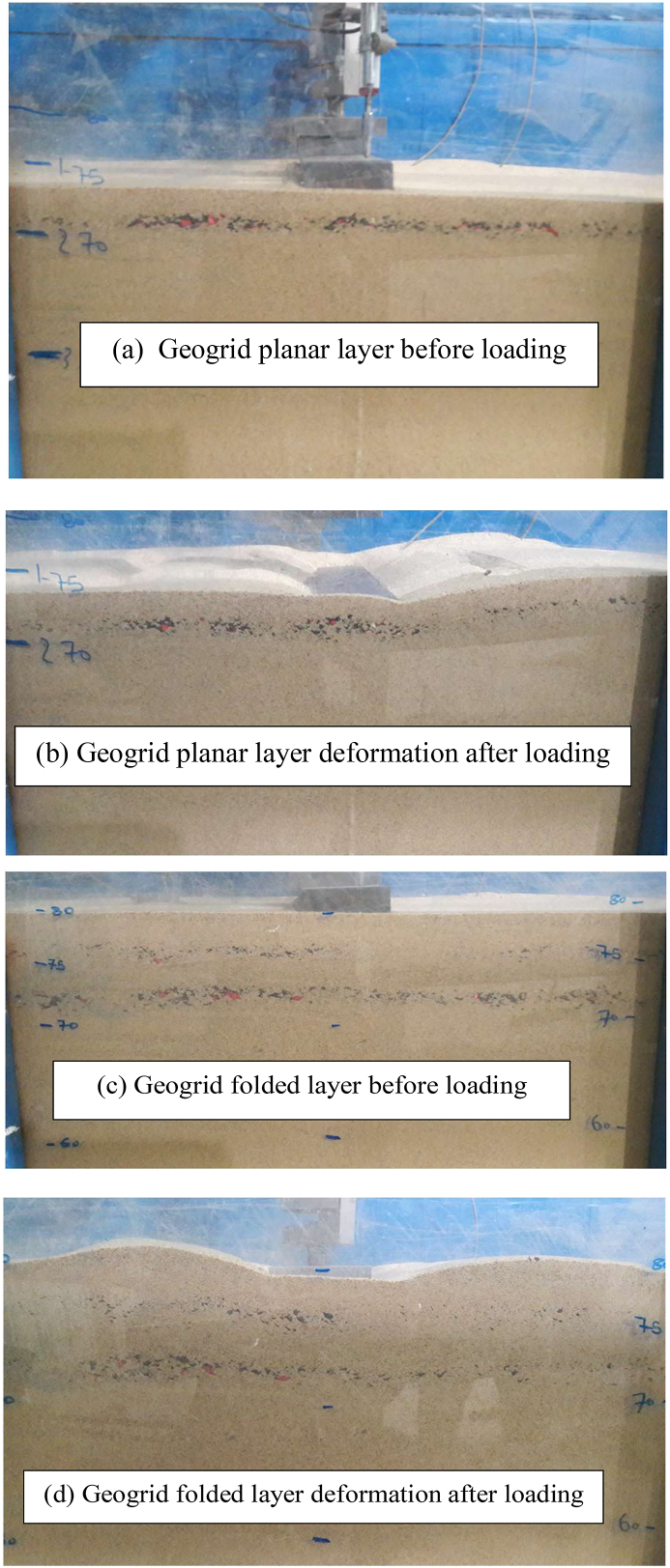
The modes of failure for (a) single planar geogrid sheet before loading, (b) single planar geogrid deformation after loading, (c) Geogrid folded layer before loading and (d) Geogrid folded layer deformation after loading.

A series of static loading tests have been conducted on a fine sand-tire shred mixture to highlight the influence of a novelty geogrid wraparound method different from the geogrid planar technique. In the planar technique, reinforcement geosynthetic materials are usually placed horizontally in the foundation [1, 9, 11, 16, 25, 34, 35, 36, 43, 44]. On the contract, in the wraparound method, a part of the geogrid sheet was wrapped around the end condition with lap lengths of *l* = 0.25L and 0.5L (where L is the original length of geogrid sheet) and inserted within the soil foundation at various embedment depths. In addition, the geogrid was completely folded with an overlap length (*l* = 0.75L and 1.0L). Based on the findings of the literature studies [14, 15, 16, 17, 18], a small increase in carrying capacity with the inclusion of a planar geogrid sheet with a length of L > 5 B was reported. Therefore, the findings of the experiment in horizontal geogrid planar form (L/B = 5) were used for comparison with the findings of the novel technique. To prepare the wraparound geogrid sheet, using length of L/B = 5 (i.e., L = 50 cm) at an embedment depth of u/B = 0.3 (i.e., u = 3 cm) is put horizontally within the soil bed. The result of this test proves a significant increase in carrying capacity due to the mobilization of tensile strength at the soil-geogrid interaction. In addition, the distributions of vertical stresses are distributed over a larger length of folded geogrid and spread to greater depths inside the soil bed. However, as the applied pressures increase, the settlements of the strip footing increase. As a result, the folded geogrid sheet is laid at the uppermost depth (d/B = 0.2 and u_f_/B = 0.3) by wrapping a part of the geogrid sheet around the end condition with the overlap length *l*/L = 0.25 (i.e., *l* = 12.5 cm). Similarly anticipated, the mobilized frictional strength between the lap component element of the wraparound geogrid sheet and the soil grains leads to an improvement in the carrying capacity of the reinforced soil. The failure happens as a result of a local punching-shear test, but this increase is insufficient since the soil grains travel upward from the two sides of the footing. To solve this issue, the sheet of geogrid can be wrapped on both sides at the bottom of the footing with an overlap length of *l*/L = 0.5 (i.e., *l* = 25 cm) and included at the embedded depth of d/B = 0.2 (i.e., d = 2 cm) and u/B = 0.3 (i.e., u = 3 cm). As a result, the carrying capacity improves greatly, and the reduction of settlement ratio is large enough. When the overlap length is used to be larger (*l*/L = 0.75 i.e., *l* = 37.5 cm and *l*/L = 1.0 i.e., *l* = 50 cm) and connected two ends of the overlap length are shared with others, the carrying capacity has improved high significantly and the settlements are reduced more smaller than those obtained with using the horizontal planar geogrid sheets. Due to the fact that two of the overlap length sides of folded geogrid are shared and fixed with them, the geogrid sheet is subjected to an extra tensile tension and additional lateral strength, which improve the load-settlement behavior of strip footing resting on reinforced sand. Experimental results of the mechanisms of modes failure are shown in Figure 6.

The illustrations of planar and folded geogrid layers before and after the plate load test are described in Figure 6 (a, b, c and d). By comparing the shape of the geogrid sheet inclusion before and after the loading test, the geogrid deformation shape after loading can be displayed, as shown in Figure 6. After each plate load test, the width of punching failure on the surface of the geogrid sheet is measured. A large punching width in the geogrid planar sheet was measured, as demonstrated in Figure 6b. Therefore, the failure modes of reinforced soil with the geogrid planar sheet can be predicted. On the other hand, a small width was measured using a geogrid folded sheet after the loading test, as displayed in Figure 6d. As a result, a large progressive of slip line is formed underneath geogrid folding. This is because the wraparound geogrid performs as a piece of the foundation when the external load is applied to the strip foundation. A detailed investigation of the wraparound geogrid embedded in fine dense sand under the strip footing susceptible to load is required to explain the maximum bearing capacity in the whole folded geogrid condition. Due to lateral expansion, the breadth of a folded geogrid might expand by several centimeters. The earth inside the geogrid sheet also seems to densify and become more compact, as if it were bonded to the foundation. Due to the expansion of the reinforcement, any external force exerted on the foundation causes a tensile force to be applied to the geogrid layer as well. The folded geogrid sheet restrained soil particles to increase the vertical effective stress.

**Figure 7:**
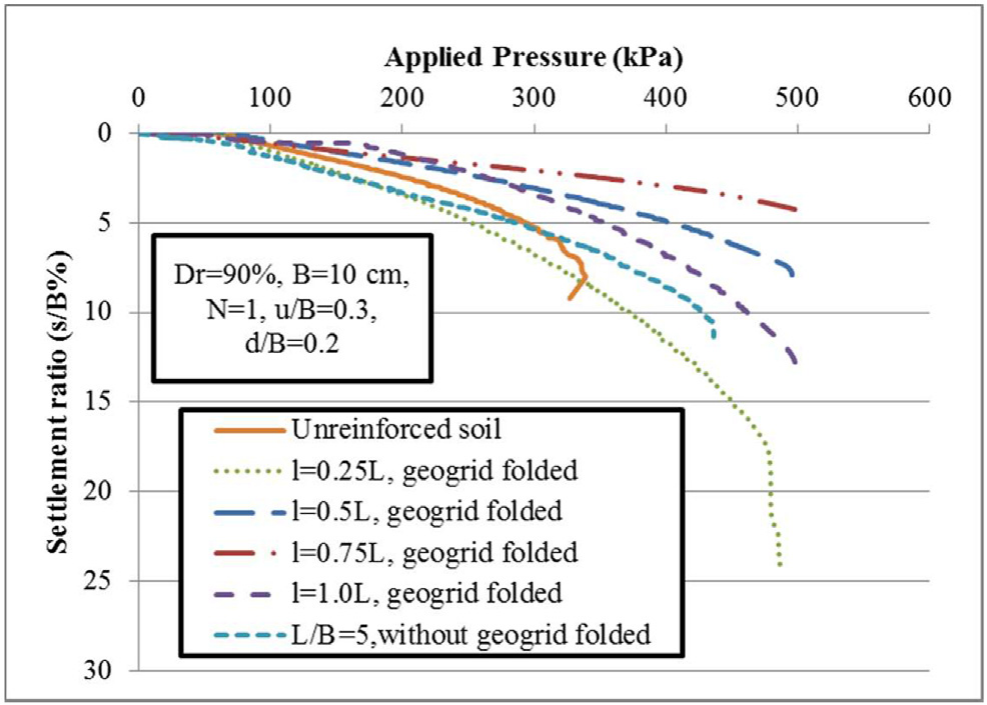
Pressure-settlement behavior of a single folded geogrid sheet for different lap lengths.

Figure 7 displays the findings of the plate load test (PLT) observational study describing the settlement ratio (%) of the strip foundation concerning the applied load per unit surface. Respecting to both Figure 7 and Table 3, the BCR_2%_ and BCR_10%_ ratios of the mixture of the tire shred-soil foundation are 4.33 and 1.7 for one planar geogrid layer (u/B = 0.3, L = 5B), 7.18 and 2.22 for one folded geogrid sheet (*l* = 0.5 L), 10.25 and 2.23 for one folded geogrid sheet with (*l* = 0.75 L), and 7.18 and 2.26 for one folded geogrid sheet (*l* = 1.0 L). Figure 7 shows that at lower settlement ratios, the load-carrying capacity of reinforced and nonreinforced soil exhibit inelastic behavior. At larger settlements, the soil without geogrid reinforcement fails at a lower bearing capacity. This means that shear mode failure controls the load-settlement behavior of nonreinforced soil. With the inclusion of geogrid sheets, the carrying capacity of reinforced soil increased with an increased settlement. The larger settlement refers to the stiffness and reaction modulus of the reinforced soil being greater than the nonreinforced soil status. Therefore, by using the geogrid sheet, the load-settlement response of dense sand underneath strip footing is improved.

**Table 3:** Findings of the plate load tests of strip footing in both nonreinforced and reinforced soils.

Status of reinforcement	*l*/L	u/B	d/B	BCR_2%_	BCR_10%_
Only tire shred (without geogrid)	-	0.3	-	5.38	2.12
With geogrid folded	0	0.3	0.2	4.3	2.14
With geogrid folded	0.25	0.3	0.2	4.35	2.18
With geogrid folded	0.5	0.3	0.2	7.18	2.22
With geogrid folded	0.75	0.3	0.2	10.25	2.23
With geogrid folded	1	0.3	0.2	7.18	2.26
With geogrid planar	-	0.3	-	4.33	1.70

Therefore, the proposed novel method has a great influence on the settlements, deformation, and relative displacement of the soil-footing interaction system. Therefore, the grain particles of sandy soil within the folded geogrid sheet have no relative movement to the foundation. Similarly, a wedge-shaped failure region is created under the folded geogrid sheet. It appears that using this new technique has connected with the shallow strip foundation to form a bigger breadth and greater embedment depth. This explanation observed the impact of the additional confinement carrying capacity due to the introduction of the wraparound geogrid sheet. Table 3 presented the findings of the strip footings for the width of the footings of B = 10 cm in both nonreinforced soil and folded geogrid reinforced soil.

#### 3.2.2 Impact of embedment depth of semifolded geogrid sheet

**Figure 8:**
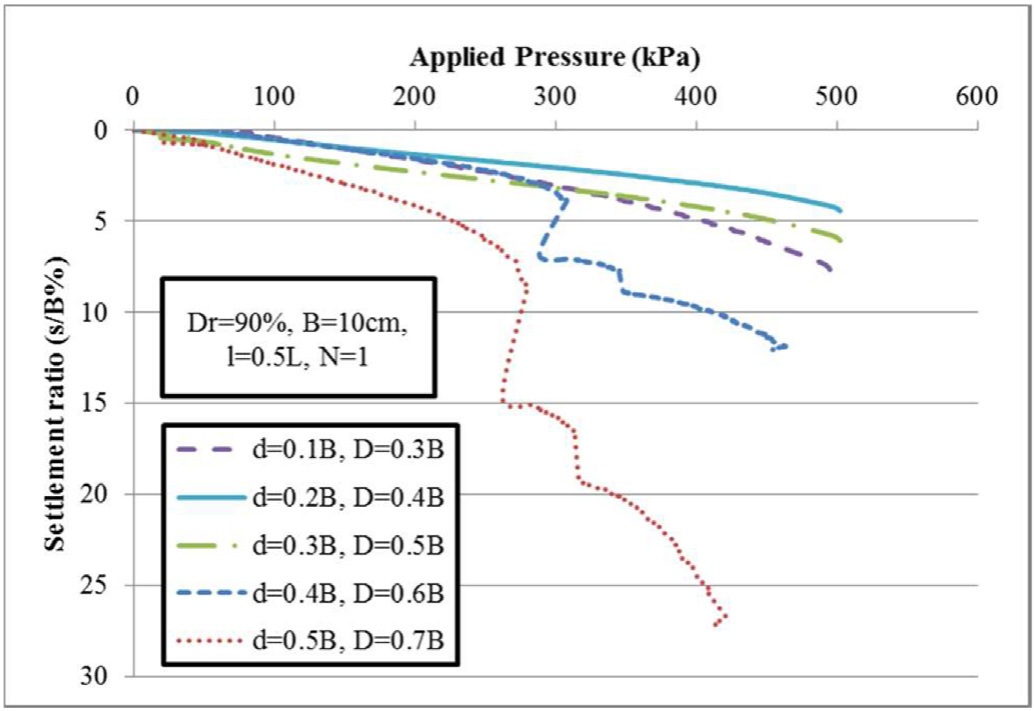
Pressure-settlement behavior of a single-folded geogrid sheet for different embedded depths.

In reinforced soils with semifolded geogrids, the embedding depth of reinforcement sheets is an important parameter. Different findings were studied about parameter (u) for planar geogrid sheet resting underneath strip load tests. The previous researchers have concentrated about the critical magnitudes of uppermost embedment depth (u) beyond which a further increase does not affect the carrying capacity, as described in the previous section. For the folded geogrid sheet, five various embedment depth ratios (d/B = 0.1–0.2- 0.3- 0.4 and 0.5) from the foundation base are tested for the upper part of overlap length of *l*/L = 0.5 of the single folded geogrid sheet (where the total uppermost embedment depths are considered in the dimensional condition u/B = 0.3, 0.4, 0.5, 0.6 and 0.7). The proposed thickness of the folded geogrid sheet denotes as x/B = 0.2 (i.e., x = 2 cm). In which the soil relative density and footing width are 90% and B = 100 mm, respectively. The findings of the applied pressures against the settlement ratios of the single semifolded geogrid sheet are shown in Figure 8. The results indicated that the embedment depth ratio of u/B = 0.4 provides the greatest ultimate carrying capacity and the lowest settlement values.

The test results are inferred that using the wraparound geogrid sheet have significant effect due to a great increase in ultimate carrying capacity when placed within fine dense sand soil at total embedment depth ratio D/B = 0.4. The confinement effect induced by the folded approach is mostly responsible for the improvement of load-settlement behavior [37, 38, 39, 40, 42]. Therefore, geogrid sheets should usually use to be inserted within dense to very dense sand with appropriate semifolded geogrid sheets to achieve the highest performance due to the increase in the subgrade reaction modulus and ultimate carrying capacity. It should also be noted that this simple technique requires a relatively smaller plot width to build the reinforced soil bed.

When using one geogrid folded sheet at embedment depths D/B = 0.6 and 0.7, the load-settlement response of the shallow strip footing shows a nonlinear zigzag curve. A reduction in the bearing pressure will also result in an increased settlement. As such, in this state, the failure mode induced within sandy soil will spread outward from the footing. Whenever the shallow footing is under pressure equal to q_ur(1)_, sudden jerks will accompany foundation movement [41]. When the shear bond lines of failure surface extended to the horizontal ground surface, the foundation must move significantly in the soil. It is observed the performance effect of the upper part of the semifolded geogrid sheet (i.e., overlap effect), where ruptures occur at the transverse and longitudinal ribs. In addition, due to the inclusion of a semifolded geogrid sheet, an increase in bearing pressure will be occurred with a larger increase in strip footing settlement. This behavior indicates to the basic role of the lower part of the semifolded geogrid sheet. The load-bearing capacities of the strip footing with inclusion of one geogrid folded sheet at total depths D/B = 0.6 and 0.7 of q_ur(1)_ = 310 and 300 kPa, are referred to as the first failure bearing pressure at small settlement ratios of 4% and 8% [41]. Note that the second peak bearing pressure values, q_ur(2)_ = q_ur_ = 350 and 325 kPa are represented to the mode of local shear failure with inclusion of semifolded geogrid in reinforced soil at the settlement ratios of 7.5% and 16%, respectively, as demonstrated in Figure 12. The third peak bearing capacities are 460 and 440 at larger settlement ratios of 12.5% and 26%, respectively. Therefore, at a deeper embedment depth, the effectiveness of the folded geogrid sheets is negligible. This behavior is similar to the behavior of the strip footing with a planar geogrid layer that is included at a large depth under a strip footing. Consequently, a failure mode occurs upon the uppermost geogrid-reinforced sheet, as reported by Binquet and Lee [1].

#### 3.2.3 Impact of the thickness of the semifolded geogrid

**Figure 9:**
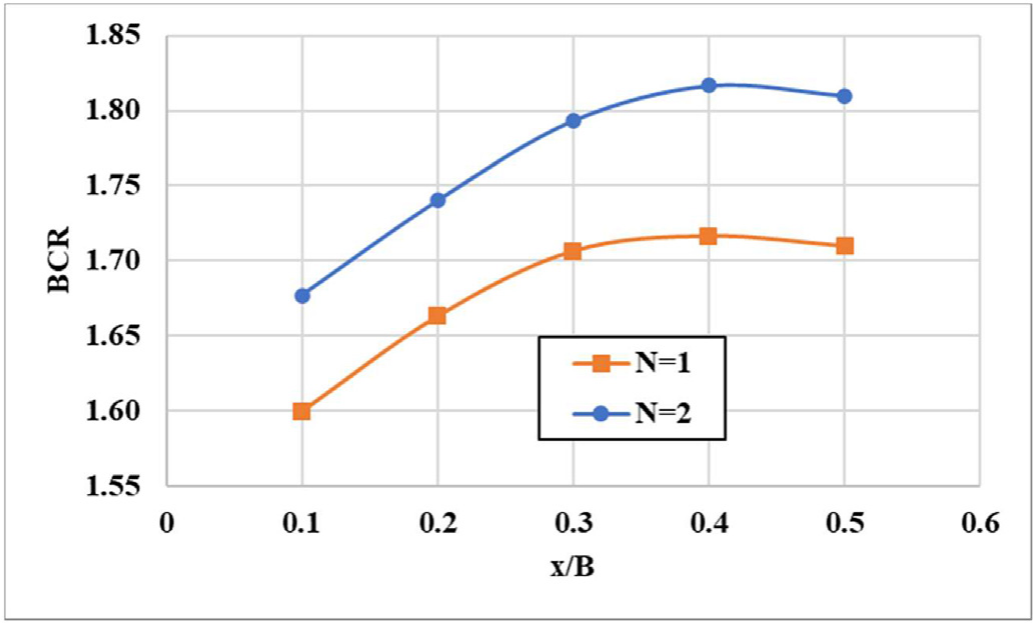
Variation in bearing capacity ratios (BCRs) with thickness (x/B).

In Figure 9, bearing capacity ratios are compared for a semifolded geogrid sheet (5B) with a similar embedment depth (u = 0.2B), but with various geogrid sheets (N = 1 and 2). A comparison shows that the increased of number of semi wraparound geogrid layers impacts the soil bed final carrying capacity significantly. The sandy soil final carrying capacity increases as the number of semifolded geogrid sheets increases. The optimum thickness (x) for semifolded geogrid layers has been indicated to be 0.35–0.45 of the footing width (B) for the higher load-carrying capacity ratios with reinforcements layers N = 1 and 2. Using fully wrapped geogrid sheets, bearing capacity ratios increased almost nonlinearly until an average thickness of x/B = 0.4 was reached. After that, there was no further improvement. As for enhancing the carrying capacity, it was found that the semiwrapped geogrid sheet behaves similar to the geocell reinforcement with a greater confinement effect that was responsible for increasing the lateral strength of the sandy soil. In semiwrapped models, the three-dimensional confinement effect can be represented as a centrally pressed beam or slab when compared to frictional resistance along a geogrid. Since the geogrid reinforcement is semifolded, it behaves as a coherent body and deforms as a slab or beam under the effect of axial tension instead of being a planar layer. In addition, Latha et al. [13], Avesani-Neto et al. [19], Biswas et al. [21], and Zhang et al. [49] have been obtained similar findings for geocell-reinforced soil bed.

#### 3.2.4 Effect of multilayer semifolded geogrids

**Figure 10:**
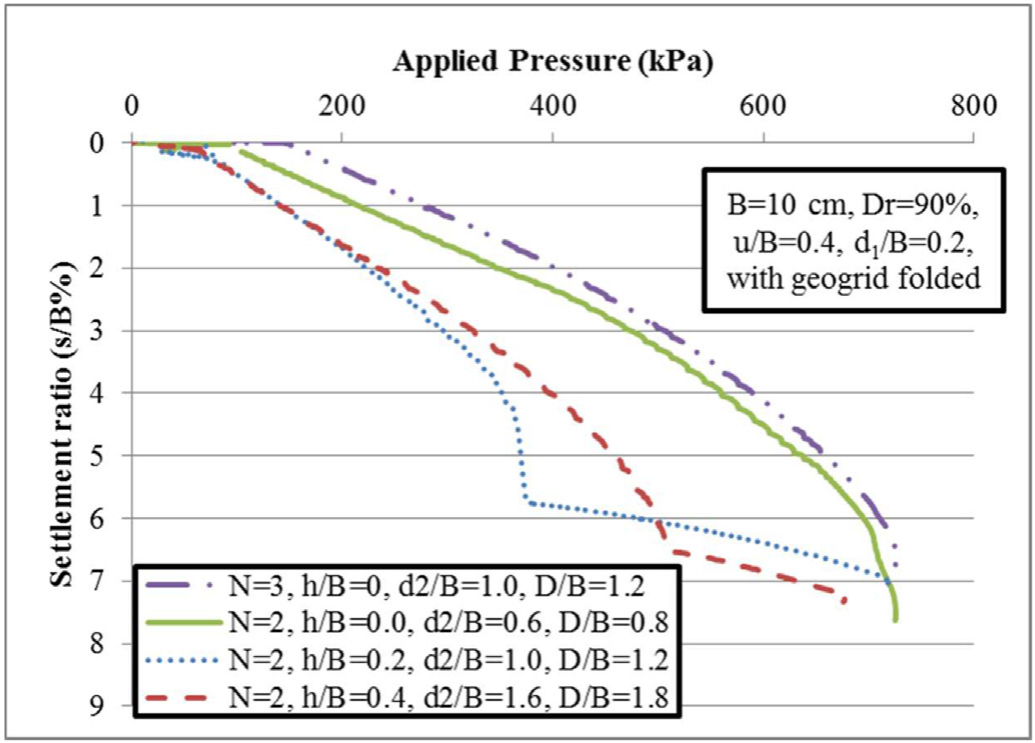
Behavior of a multi folded geogrid sheets for different vertical spacing ratios (h/B).

A model was developed using ideal u/B, d_1_/B, *l*/L_1_, and Dr values of 0.40, 0.2, 0.5, and 90% to determine the impacts of vertical spacing ratio between semifolded geogrid sheets (h/B). The total depth of embedment any geogrid sheet denotes as (D = u+ (h + x) ×N, where u = d_1_+x and d_2_ = u + h). In which both d_1_ and d_2_ represent the embedment depths of the folded lap component for the one and two folded geogrid sheets, respectively. The details of the layout are displayed in Figure 5. The curves of bearing pressure-settlement ratio are presented in Figure 10 with consideration the variety values of embedment depths h/B. It can be observed that by the increasing carrying capacity of the reinforced soil, the optimum ratio of vertical spacing between folded geogrid sheets is determined at h/B = 0 for Dr = 90 and gives a greater bearing capacity ratio of BCR_*f*_ = 4.42 for two and three geogrid sheets. Figure 10 displays the variation of applied pressures with the equivalent displacement at the given relative density (Dr) of 90%. The curves reveal a peak point for h/B = 0 for the two and three folded geogrid sheets since the geogrid sheets have induced greater tensile strengths and relative displacements that leading to occur failure. It refers that the strains and deformation induced at the mobilized tensile strength of the folded geogrid sheet is lower than strains at the final load-carrying capacity. Moreover, the findings demonstrated no significant difference between the curves at the elastic stage of the first loading (settlement ratio s/B% is lower than 2%) for folded geogrid sheets with vertical distance ratios of h/B = 0.2 and 0.4; nevertheless, the distinction between curves was more evident at larger settlement ratio (i.e., s/B% is larger than 4%). When h/B = 0.0 (without any vertical spacing between folded geogrid sheets), the magnitudes of settlement ratio, confining pressure, and carrying capacity denote at lowest, highest, and greatest magnitudes, respectively. Furthermore, it was discovered that increasing the vertical depth increases the confining pressure; regardless, no significant discrepancy was discovered for an embedment depth ratio larger than zero i.e., h/B > 0.

For two folded geogrid sheets that are placed at the embedment depth ratios D/B = 1.2 and 1.8, the sudden reduction in the load-settlement behavior that explains the sudden of failure mode occurs at a certain two points at the settlement ratios of 6% and 6.6%, respectively. After these sudden failure points, the hardening behavior is shown in Figure 10. This hardening behavior followed by reduction in settlement with increasing load-bearing pressure. It is explained the with the inclusion strengthens folded geogrid sheet, leading to mobilized additional passive vertical pressure and rearrangement of the effective stresses between geogrid sheets. With the inclusion of folded geogrid, the soil inside the folded geogrid layer became denser, the volume of soil was expanded and remobilized particles of sand-tyre shred around the lap length. The vertical stresses transformed from the upper geogrid folded sheet, redistributed within the depth and concentrated over the small thickness of dense sand between the two geogrid folded sheets. This leads to a sudden increase in the settlement ratios, and the soil can fail. Then, the settlement accumulates, and the load-settlement behavior of the sandy soil is improved by increasing the load-carrying capacity and reduction settlement until it reaches the ultimate point of failure mode and the failure region of the sandy soil lengthens receive to the ground surface.

#### 3.2.5 Stress and strain distributions

Because of the limited time, the stresses distributed within the soil bed and strains induced along the reinforcement sheets were measured in four states: with one planar geogrid sheet, with two planar geogrid layers, and with one and two layers of fully-wraparound geogrid.

**Figure 11:**
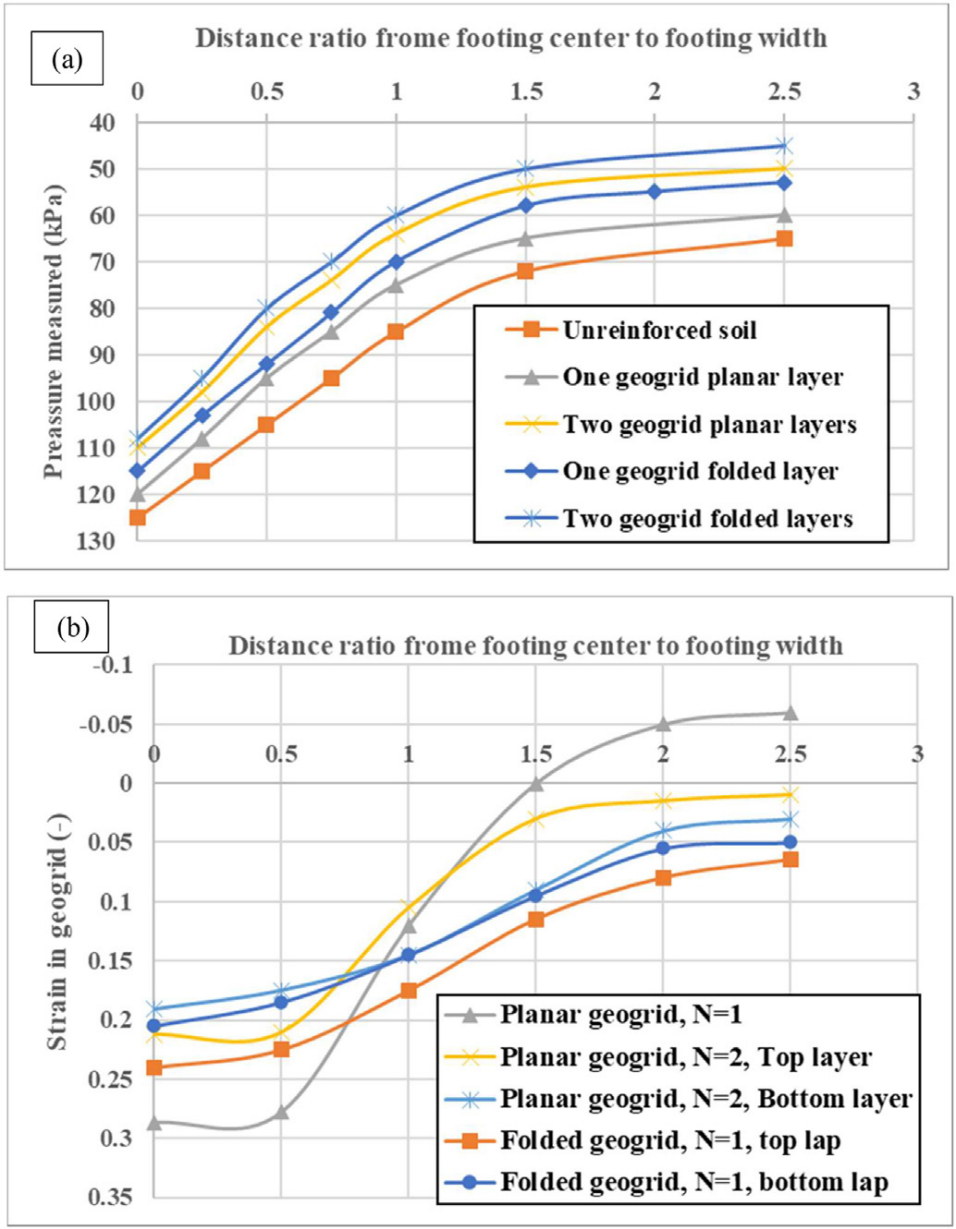
(a) Distributions of pressure measured along the geogrid sheet, (b) Distributions of tensile strain along the geogrid sheet.

In Figure 11 (a, b), the distributions of pressure measured and strain in the geogrid sheets are shown at normalized final settlements (s/B = 10%). It can be concluded that the maximum stress measured along with the geogrid reinforcement induced under the center of the strip footing decreases gradually with the spacing ratio from the footing center to the strip footing width. The greater pressures are measured under strip footing in the nonreinforced soil in comparison with reinforced states. Underneath the strip footing center, the stresses measured along the geogrid sheet are 120 kPa, 115 kPa, 110 kPa and 108 kPa for one geogrid planar, one geogrid folded, two geogrids planar and two geogrid folded sheets that are smaller than that of nonreinforced soil (125 kPa). This demonstrates that the geogrid sheet appears to reduce the stresses induced in the soil mass. This is explained by the lateral effect of the folded geogrid sheet. This reinforced zone acts as a flat slab and enables the applied load to be distributed over a larger area, which results in significant reductions in lateral displacements. As the footing settles, anyway, the soil displacements at the left and right corners of the reinforced zone increase substantially. Consequently, mobilized tensile forces are progressive in the folded geogrid sheet. Under these conditions, the tensile strength of the geogrid reached its maximum. The same results are observed for the tensile strain of the geogrid layers. The maximum tensile strain in the geogrid is induced underneath the footing center. In one planar geogrid sheet, rupture occurred in most ribs. The values of the maximum tensile strains of the geogrid for one planar geogrid, two planar geogrid layers, and one folded geogrid were 0.28, 0.22, and 0.24, respectively, when the settlement of the footing reached the ultimate value s/B% = 10. The distribution of strain along the geogrid layer is more relevant for folded geogrid sheets than for planar layers. In addition, the tensile strain of the planar and folded geogrid decreased as the number of geogrid layers increased.

**Figure 12:**
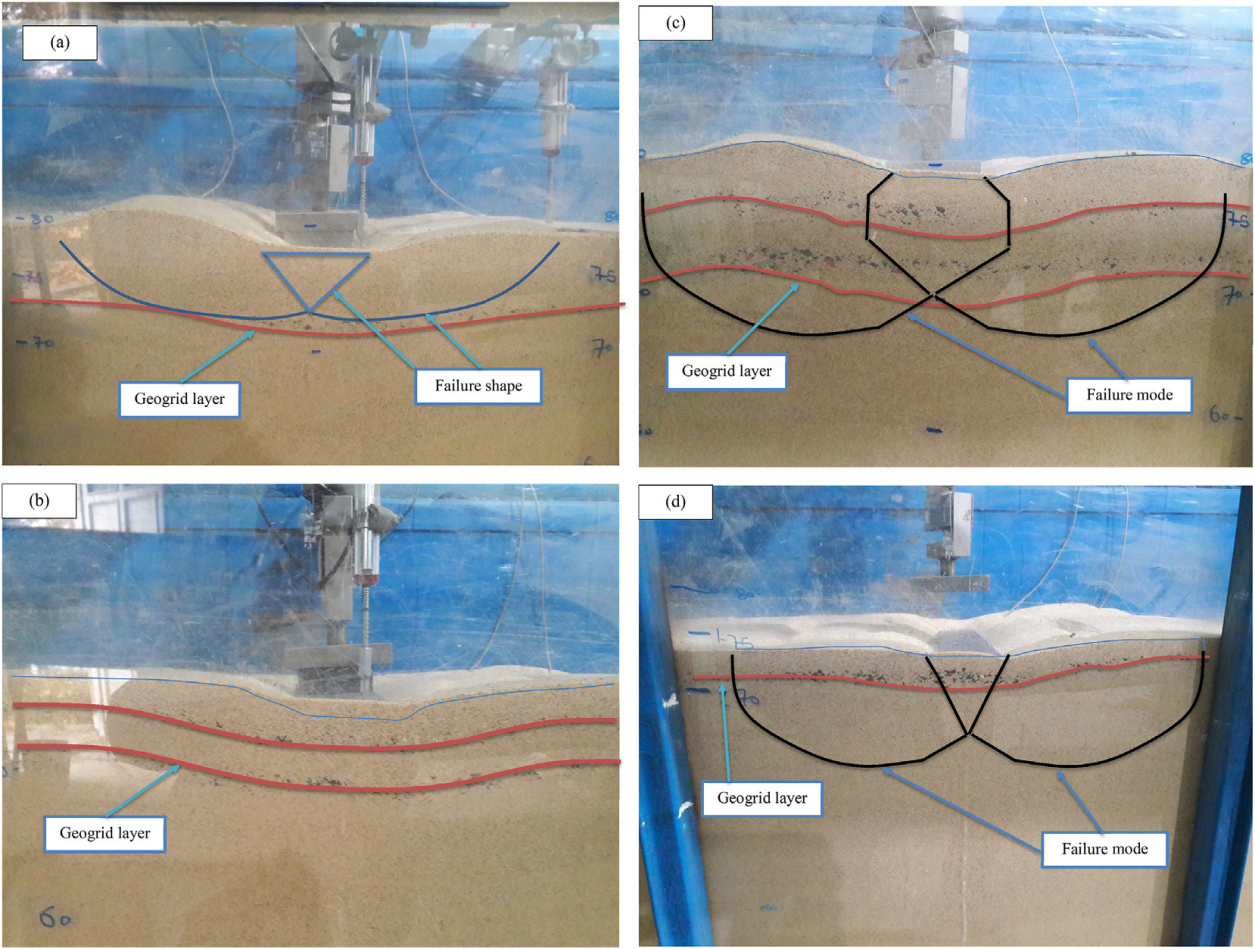
Failure mode within dense sandy soil with planar reinforcement: (a) failure upon the uppermost geogrid-reinforced sheet, (b) failure occurring analogously in a two-layered soil, (c) failure between geogrid-reinforced sheets, and (d) failure in one geogrid-reinforced sheet.

### 3.3 Release of waste tire-derived to the environment and prevention method

There are many positive aspects to used tires. Over the years, the recycling of tire scraps has increased dramatically, resulting in outputs such as grasslands, sports domains, and construction materials. Tire reuse increased from 11% in 1990 to 81% in 2017, according to the United State Tire Manufacturers Association (USTMA). However, the above number contains a significant caveat: it includes “tire-derived fuels” (TDF). Tires are burned to generate energy. The USTMA report from 2018 states that 16% of nonrecycled or nonburned tires end up in landfills. From 2013 to 2017, nearly double as many tires were disposed of in landfills. It is expected that even more tires will be dumped in landfill, as stated by USTMA.

The tire has not been redesigned in decades, but more sustainable options have recently been developed. The University of Minnesota, for instance, produced isoprene, a major component in synthetic rubber, from trees, grass, and cereal rather than fossil power in 2017. It was announced last year that Goodyear would introduce a concept tyre made from reused rubber that contains moss in the middle that soaks up carbon dioxide during release.

### 3.4 Justify the release of waste tire-derived (WTD)

The release of waste tire-derived (WTD) to the environment can be expected. It is emitted into the atmosphere in small amounts, but much larger amounts reach soils near roads and aquatic compartments. The water is more or less retained within a WTD in the case of roads supplied with a drainage technique that includes runoff cure or transportation to a WTD so that the water will not or just to a very limited extent reach surface water. Several investigations have estimated tire wear masses that are yearly discharged within surface waters, while others have made rough estimates. The emissions from German highways were assessed by Wang et al. [72] considering two strategies with various runoff rates (15 and 50%, based on [66, 67]). As part of a comprehensive study of German roads, Baensch-Baltruschat et al. [68] calculated the paths in which pollutants enter the soils and surface waters. Most of the fine WTD that is generated on roads is deposited in the soil near the road (66–76%), a less percentage about (12–20%) is discharged to surface water, and an extremely little amount approximately (2–3%) is distributed on agricultural regions. In the absence of data on soil degradation, it is presently undefined whether and how the particles will collect over time.

## 4 Mode failure

Planar and folded geogrid inclusion layers are used in the below sections to explain the mechanism of failure of shallow strip foundation resting on reinforced sand. Failure modes associated with carrying capacity types can be summarized as follows:

### 4.1 State of nonreinforced sandy soil

According to Terzaghi and Peck [45], soil failure is an extremely likely scenario that results in plastic equilibrium zones under soil loads applied to the strip footing. Experimentally, an initial outbreak occurred near the ends of footings and then slowly spread outward and downward. Through the soil above the failure surface, the development of plastic equilibrium can be observed. There is significant ground movement on both sides of the footing as a result of the failure. It is usually dense to very dense soils that fail due to general shear-type failures.

### 4.2 State of reinforced sandy soil with a planar geogrid sheet

The failure mechanisms of planar reinforcement are divided into four categories based on different studies [1, 46, 47] as follows:

(a) Failure upon the uppermost geogrid-reinforced sheet [1], as demonstrated in Figure 12a,
(b) Failure occurs analogously to a 2-layered soil, as explained by Wayne et al. [46], as displayed in Figure 12b,
(c) Failure between geogrid-reinforced sheets [46], as shown in Figure 12c,
(d) Failure within the geogrid-reinforced sheet (Sharma et al. [47], see Figure 12d.

There is a high chance of failure in one of the types (a) or (b) if the topmost layer (u) of the geogrid or the layer spacing (h) is excessive, as appears when h/B > 0.5 and u/B > 0.5. Researchers from [14, 16] have shown that to prevent these types of failure modes, the distance between the first reinforcement sheet (u) and base of strip footing, as well as the distance between reinforcement sheets (h), should be less than half the footing width (i.e., u/B < 0.5 and h/B < 0.5). When failure mode (c) occurs in the reinforced area, punching shear occurs at the strength of the reinforced region, which is higher than that of the nonreinforced soil state, and when the depth ratios (d/B) of reinforcement layer are small. After that, the unreinforced area is subjected to total failure. This mode of failure mechanism was proposed by Meyerhof and Hanna [48] firstly for the state of a strong soil resting on a weak soil bed. The solution proposed by Hanna and Meyerhof [48] was modified by Wayne et al. [46], therefore, it may be used for the computation of the foundation carrying capacity that is supported by reinforced substrates. The study by Sharma et al. [47] showed that the reinforced area had a slightly higher strength than the unreinforced base layer, while the u and h values were smaller than 0.5B, followed by the happened of failure mode within the reinforced region. Clayey and sandy soils are suitable for type (c) and type (d) failure mechanisms. A brief description of the failure patterns will be suitable along with Figure 12.

### 4.3 A state of semifolded geogrid layer reinforces soil

**Figure 13:**
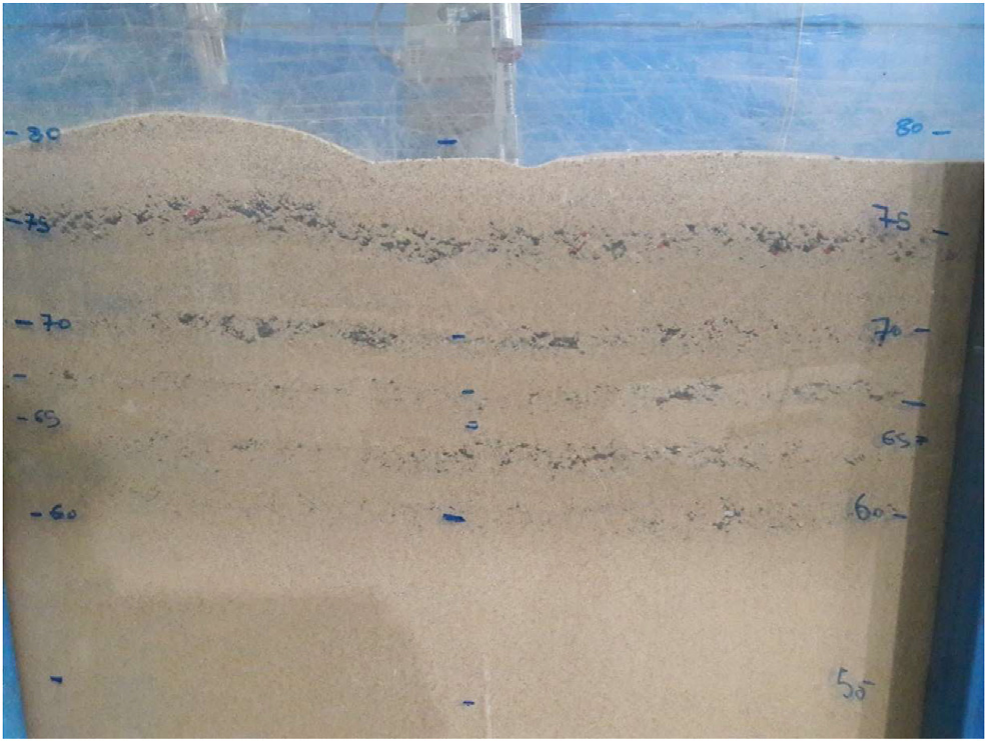
Failure mode within dense sandy soil with folded geogrid reinforcement.

Semifolded geogrids are similar to other kinds of geosynthetics that are used as horizontal reinforcement arrangements. Their load bearing capacity progressive is credited to multiple mechanisms that all work together to provide improved soil bearing capacities. Unlike planar reinforcements, geogrids folded in confinement are more efficient than planar reinforcements capable of triggering membrane and confinement effects, as shown in Figure 13. Each mechanism works in a specific way. Despite this, the mechanisms have an interrelation and were developed through the application of loading by Zhang et al. [49]. Loading causes a greater confinement effect mechanism. Geogrid folded structures increase the pressure confinement strength and stiffness of mixed sand-tyre shred material densification [50]. The tyre shred-soil mixture and the wrap-around end of the geogrid generate horizontal stresses, where mobilize an interaction shear strength between them [25]. The confinement effect is therefore exerted by two methods: improving the strength and deformability of sandy soil and dissipating the load via horizontal stresses within the geogrid. There is overlap and distribution of these stresses between the wrap-around edge mobilization of confinement passive strength of soil-tyre shred mixture and the interaction shear tension between the mixtures and the folded geogrid to form the stiffer system. In contrast to membrane effects, the relative displacements are the major effect of the mechanism. Despite this, its effectiveness is limited by the degree of interface friction strength between the folded geogrid material and the mixture of soil-tyre shred [25, 50].

## 5 Conclusion

Single shallow strip footing resting on geogrid-reinforced sand and unreinforced sand was tested under a series of static loading. There were 38 tests carried out on a small-scale physical model. The unreinforced sand foundation was tested twice, and the reinforced sand foundation was tested thirty-two times with various reinforcement configurations (planar and wraparound). During the experimental procedure, the load-settlement behavior of reinforced soil with the wraparound geogrid method is understood through the use of one type of fine sand at a relative density of 90%, one type of geogrid, one type of tire shred, strip footing width (B = 10 cm), three numbers of LVDTs, ten pressure cells, and sandboxe. Lastly, we draw attention to the following points:

1 In comparison with the installation of planar geogrid sheets, the wraparound geogrid sheets may increase the final carrying capacity of the shallow strip footing. The ultimate carrying capacity ratios BCR_*f*_ and BCR_I_ could be as high as 2.05 and 2.12, respectively, for a settlement ratio of 10%. For soil with a relative density of 90%, the highest improvement in ultimate carrying capacity occurred in the strip footing resting on soil-tire shred mixture incorporating one fully folded geogrid sheet at an embedment depth of lap element of 0.2B, using overlap length *l*/L = 1, and total embedment depth ratio D/B = 0.41.
2 By incorporating one folded geogrid sheet into the soil bed, settlement and deformation can be greatly reduced. The reduction of settlement was most effective when two sides of lap elements were shared with the inclusion of one semifolded geogrid sheet at a lap length ratio of l/L = 0.75. As a result of the interaction between soil-tire shred materials confined within the above overlap element reinforcement and folded geogrid sheet, interfacial tensile strengths result in a lower lateral strain and higher lateral confinement pressure by sharing two sides of overlaps.
3 In a fine sand-tire shred mixture with the highest relative density of 90%, using the optimum embedment depth is approximately 0.41B (in which B denotes the footing width), and the optimum embedded depth of overlap length is 0.2B to obtain the best load-settlement behavior.
4 The use of two semifolded geogrid sheets greatly increases the stiffness of the tire shred-soil mixture, improves its carrying capacity, and significantly reduces the settlement of the mixture. When the two layers were laid at optimum numbers, the highest bearing capacity ratio was obtained. Since there is no vertical space between folded geogrid sheets (i.e., h/B = 0), the BCR ratios are extremely large.

## Declarations

### Author contribution statement

Hussein Ahmad: Conceived and designed the experiments; Performed the experiments; Analyzed and interpreted the data; Contributed reagents, materials, analysis tools or data; Wrote the paper.

### Funding statement

This research did not receive any specific grant from funding agencies in the public, commercial, or not-for-profit sectors.

### Data availability statement

Data included in article/supp. material/referenced in article.

### Declaration of interest’s statement

The authors declare no conflict of interest.

### Additional information

No additional information is available for this paper.
